# Realistic Simulation for Body Area and Body-To-Body Networks

**DOI:** 10.3390/s16040561

**Published:** 2016-04-20

**Authors:** Muhammad Mahtab Alam, Elyes Ben Hamida, Dhafer Ben Arbia, Mickael Maman, Francesco Mani, Benoit Denis, Raffaele D’Errico

**Affiliations:** 1 Qatar Mobility Innovations Center (QMIC), Qatar Science and Technology Park (QSTP), P.O. Box 210531 Doha, Qatar; elyesb@qmic.com (E.B.H.); dhafera@qmic.com (D.B.A.); 2 CEA-LETI, Minatec Campus, 17 rue des Martyrs, 38054 Grenoble, France; mickael.maman@cea.fr (M.M.); Francesco.MANI@cea.fr (F.M.); benoit.denis@cea.fr (B.D.); raffaele.derrico@cea.fr (R.D.)

**Keywords:** body area networks (BAN), body-to-body networks (BBN), deterministic channel and mobility modeling, semi-deterministic channel modeling, realistic simulation, accurate mobility and radio link modeling, IEEE 802.15.6 Standard

## Abstract

In this paper, we present an accurate and realistic simulation for body area networks (BAN) and body-to-body networks (BBN) using deterministic and semi-deterministic approaches. First, in the semi-deterministic approach, a real-time measurement campaign is performed, which is further characterized through statistical analysis. It is able to generate link-correlated and time-varying realistic traces (*i.e.*, with consistent mobility patterns) for on-body and body-to-body shadowing and fading, including body orientations and rotations, by means of stochastic channel models. The full deterministic approach is particularly targeted to enhance IEEE 802.15.6 proposed channel models by introducing space and time variations (*i.e.*, dynamic distances) through biomechanical modeling. In addition, it helps to accurately model the radio link by identifying the link types and corresponding path loss factors for line of sight (LOS) and non-line of sight (NLOS). This approach is particularly important for links that vary over time due to mobility. It is also important to add that the communication and protocol stack, including the physical (PHY), medium access control (MAC) and networking models, is developed for BAN and BBN, and the IEEE 802.15.6 compliance standard is provided as a benchmark for future research works of the community. Finally, the two approaches are compared in terms of the successful packet delivery ratio, packet delay and energy efficiency. The results show that the semi-deterministic approach is the best option; however, for the diversity of the mobility patterns and scenarios applicable, biomechanical modeling and the deterministic approach are better choices.

## 1. Introduction

With the continuous exponential rise of wearable devices and applications, it is anticipated that by 2019, there will be more than 150 million wearable devices worldwide [1]. While fitness and healthcare remain the dominant wearable applications, other applications include fashion and entertainment and augmented reality, and rescue and emergency management are emerging, as well [2]. Wireless body area networks (BAN) are an implicit and well-known research discipline, which fosters and contributes towards the rapid growth of wearable technology.

Concerning the BAN modeling, there are several methods and approaches, including real experiments, analytic analysis and network simulations [3,4,5,6,7,8]. Every approach has pros and cons, for example mathematical derivations can be developed to model specific behavior, constraints and optimization [5,6]. However, due to many assumptions in the analytical derivations, the models often are not realistic. Further, while modeling multiple functionalities, the mathematical models become very complex. To develop analytical expressions for diverse BAN applications (having space and time channel variations, dynamic mobility patterns and varying radio links), the resultant models are highly complex and difficult to develop [9].

Another approach is to execute measurement campaigns by deploying various antennas on the body to communicate between sensors and coordinating device [6,7,8]. Such measurement campaigns help to acquire real-time data, which can be transformed to mathematical models and statistical distributions. However, there are a number of limitations, especially to realize measurements for dynamic mobility, which are critical to various applications. Further for the applications, where body-to-body communication is important, large-scale deployment is required, which is often not feasible. Finally, there are also storage and processing constraints with miniaturized wearable devices to handle real-time data and processing.

Network-level packet-oriented simulation is another choice with a key advantage that an extensive analysis can be performed by having many variations of the parameters and models from all of the layers of the communication and protocol stack. Consequently, with a large-scale, extensive and realistic performance evaluation of the new approaches, algorithms can be achieved.

Concerning the BAN simulators, there is more need for realistic simulators than before. For example, in healthcare and fitness with the possibility to deeply analyze medical signals, new signal processing algorithms and techniques based on real data are rapidly emerging [10,11,12]. Such new approaches and algorithms need to be evaluated at the network scale by taking into account the impact of the communication and protocol stack. This requires a realistic and cross-layer BAN-specific packet-oriented network simulation environment.

With regards to existing BAN-specific simulators, there are a few options. For example, OMNet++ and its associated frameworks, such as Castalia [13] and MiXiM [14], have some BAN-specific modules. Castalia relies on a real-time measurement campaign to develop channel models, which include space and time variations and the impact of body shadowing and fading [15]. However, the measurements conducted are limited to certain scenarios and applications setups. It considers line mobility, which is not realistic for the diverse mobility patterns of emerging applications [2,16]. Whereas, MiXiM provides BAN-specific mobility models [17], which mimic mobility structures, including the modeling of posture variations and temporal correlation. With the addition of these mobility models in MiXiM, it is a viable simulation environment for the simulation of PHY and MAC layers. However, since 2011, there has been many new applications, and some are still emerging, where body-to-body (B2B) communication is inevitable [18,19,20]. To the best of our knowledge, MiXiM mobility models are still based on [17], which is not developed for body-to-body networks (BBN). In addition, there is hardly any simulator that is compliant with the BAN-specific standard (*i.e.*, IEEE 802.15.6). 

### Contributions

There are great opportunities and possibilities to develop a realistic BAN and BBN simulator, which can provide a realistic simulation environment and platform. To meet the requirements of the emerging new applications, this work presents a complete all-in-one package for modeling and evaluating body area networks and body-to-body networks. This includes realistic channel models based on a measurement campaign for both BAN and BBN, IEEE 802.15.6 standard-complaint modulation, path loss, radio link models at the physical layer, carrier sense multiple access collision avoidance (CSMA/CA), scheduled access MAC protocols, as well as random channel and time-shared co-channel interference strategies, which are evaluated and are available at the MAC layer. All of these models are standard specific and provide a benchmark for BAN- and BBN-specific future algorithms’, strategies’ and protocols’ realistic performance evaluations. Further, the performance of IEEE 802.15.4 under co-located BANs is also a part of the package.

In this paper, we present full deterministic and semi-deterministic (statistical) mobility approaches for BAN and BBN. The full deterministic approach is an enhanced version of the IEEE 802.15.6 standard proposed channel models by providing dynamic, space and time channel variations. The model relies on motion capture systems and biomechanical mobility modeling to have diverse mobility patterns. On the other hand, the semi-deterministic approach relies on a real-time measurement campaign and statistical modeling, which provides more realistic channel characterizations (*i.e.*, link correlated and time-varying on-body and body-to-body models). We compare these approaches against the IEEE 802.15.6 standard proposed channel models. The performance is evaluated through several metrics, including the packet delivery ratio (PDR), energy consumption and packet delay. The results show that the semi-deterministic approach is more realistic under a constraint network topology and simulation setting, which is a limitation for a fair comparison. However, for the diversity of the mobility patterns and scenarios, biomechanical modeling and the deterministic approach are better choices.

The rest of this paper is organized as follows. After this Introduction, related works about existing simulators, their potential benefits and shortcomings are highlighted. This is followed by the proposed simulator, for which, first, the architecture is introduced, then the two mobility and channel modeling approaches are proposed. Finally, a case-study is presented that compares the two approaches, and the results are presented.

## 2. Related Works, Simulators and Theirs Limits

In this section, first, we present briefly the existing well-known network simulators. Then, BAN-specific frameworks are explored, and finally, a summary is presented to highlight the limitations.

### 2.1. Classical Wireless Network Simulators

There are a number of network simulators that can be found in the literature. This includes NS-2 [21], NS-3 [22], OPNET [23], OMNeT++ [24], WSNet [25], *etc*.

The NS-3 simulator is an open-source and discrete-event network simulator, which was first developed in 2006 [22]. The recent version NS-3.24.1 was released on 15 September 2015 [26]. NS-3 is not an extension of NS-2. Both simulators are written in C++, but NS-3 is a new simulator, which does not support the NS-2 APIs. Concerning the BAN studies, using NS-2 and NS-3, there are a few studies, such as [27,28]; however, these studies are based on unrealistic BAN channel models, which impact the interference evaluation. OPNET is another widely-used general wireless network simulator [23]. The latest version of the OPNET modeler was released in November 2015 [29]. BAN-specific coexistence is studied in [30,31]; however, the simulator does not consider the peculiarities of BAN propagation. OMNeT++ is an extensible, modular, component-based simulator in which a number of libraries and frameworks are available. The most relevant frameworks for BAN are MiXiM [14] and Castalia [13], as explained in Section 2.2.2. MiXiM is an OMNeT++ modeling framework for mobile and fixed wireless networks. MiXiM concentrates mainly on the physical (PHY) layers of the protocol stack and offers detailed models of radio wave propagation, interference estimation, radio transceiver power consumption [32] and mobility models. In addition, the integration of various mobility models (*i.e.*, MoBAN [17]) makes it a more realistic option to simulate low layers (*i.e.*, PHY and MAC). Finally, WSNet [25] is another event-based packet-oriented network simulator, which is used for node and environment simulation. The simulated nodes are built with a hardware component, a software component or the behavior/resource of the node. One of the key advantages of using the WSNet simulator is that there is an associated node platform simulator, WSIM [33], which allows simulating different components of the sensor nodes. Regarding BAN-specific works, traffic-aware dynamic MAC for BAN is implemented in WSNet; further, convergence behavior is heuristically modeled and simulated in WSNet [34,35]. However, the protocol is evaluated under unrealistic channel and mobility models. Please note, similar studies in the future can be evaluated more realistically after having all of the modules presented in this work. Mo recently, a physical simulator, PyLayers [9], has been interfaced in real time with WSNet. This open source project addresses indoor radio propagation and provides a site-specific ultra-wide band-oriented simulator for BANs. This new framework will be discussed in Section 2.2.2.

### 2.2. BAN-Specific Frameworks

In this section, we present BAN-specific frameworks to highlight the BAN-specific contributions in various simulators discussed above. In particular, it includes BAN-specific wireless channels and networks frameworks.

#### 2.2.1. Channel-Oriented Frameworks

Several categories of BAN communications can be defined according to the specific node positions. These differences yield to a large variety of channel models that have been proposed in the literature. As our study focuses on BANs’ simulation, we will address on-body channels for intra-BAN communications and body-to-body channels for modeling the interference generated by one BAN on another and the inter-BAN communications.

Investigations on the on-body channel have been carried out for several years. Initially, works attempted to classically model the channel as a function of the distance between the on-body antennas [36]. The same approach was used in the IEEE 802.15.6 reference document for the channel, yielding the channel model (CM3-A) and channel model (CM3-B) [37] (as detailed later in Section 3.2.2). Later, a scenario-based approach [38] has been found more suitable to describe such channels as body shadowing and human mobility. It is found that inter-node distances are not the only factors that have an impact to the greatest extent. Therefore, a scenario can be represented as the specific Tx and Rx antenna location on the body along with the environment and human body mobility condition.

With respect to the on-body channel, body-to-body channels have been less investigated. In the IEEE 802.15.6 model, CM4 has been proposed for these emerging communications, especially focusing on hospital environments [37]. In the framework of cooperative communications, classical indoor and outdoor environments and different dynamic conditions of the users have to be considered [39,40]. Body-to-body scenarios have been investigated to characterize interference given by nearby BANs at 2.4 GHz and 60 GHz [41].

Aiming at the exploitation of the body-to-body cooperative communications, channel measurements were performed at 2.45 GHz in [42]. Different movements were considered for different subjects: forward walk, opposite walk, parallel walk and rotation. The results show that the mean channel gain is strictly related to the specific node emplacement (relative position of the transmitter and the receiver) and to the antenna type considered. Shadowing from the body is a predominant effect. The NLOS condition given by the obstruction from one subject implies an important reduction of channel gain. The direct path being strongly attenuated, all propagation comes from multi-paths on the surrounding environment.

In [43,44], the authors propose a simulation platform to study interference between BANs and propose to take advantage of possible inter-body channel correlations to judiciously select the slot in the superframe. This platform is interesting, but it needs a more accurate inter-BAN channel model (scenario-based instead of statistical). In [45,46], Dong and Smith evaluate the co-channel interference of multiple BANs using a TDMA MAC using a guaranteed time slot with a realistic environment model. Their simulations are based on large sets of empirical everyday activity channel data, but no network simulators are linked to this database.

Finally, context-aware body area networks (CABAN) [47,48] introduce channel fading characteristics for an interactive environment. For example, [47] proposed a context-aware measurement system, which is capable of simultaneously characterizing both off-body and on-body channels. It is reported that fading characterization was often arbitrary due to the divergence of the empirical data to that of the fading distributions and that reflects that with context variation and mobility, the channel variations can be significantly different.

#### 2.2.2. Network-Oriented Frameworks

The authors in [49,50] present a System-C-based fast simulator that can provide accurate timing and energy estimation for health monitoring applications for BAN. This simulator is able to obtain timing and energy measurements for each function in the program, as well as for each module in the hardware, which is then used for the optimization towards critical functions and/or components. The authors in [51,52] present another System-C simulator using a joint mobility and semi-deterministic BAN channel model with a spatial and temporal dependency based on ray tracing techniques and benchmarked with a measurement campaign. This simulator and its BAN dedicated protocol in [53] have also been validated through the implementation and tests on a real platform.

To create a complete simulation tool for BANs, PyLayers [9] has been interfaced in real time with WSNet. The idea is to simulate in real time the PyLayers mobility feature with statistical radio channel models (PHY layer) and WSNet (upper layers), calculating the performance of the simulated scenario. The simulator embeds several modules, including: an agent mobility simulator based on a discrete event simulator to which a multi-cylinder body model can be associated; a deterministic channel propagation simulator based on a ray tracing approach for body-to-body and off-body communications; a statistical model for on-body communications taking into account the environment and the body shadowing effects. Nevertheless, in this co-simulation, the resultant models are highly complex and difficult to develop. In this regard, the realistic channel, mobility and radio link models introduced later in Section 3 can provide a realistic platform for BAN-specific performance evaluation and comparative studies for novel algorithms and protocols.

In Castalia, NICTA [54] has contributed to developing BAN-specific wireless channels, mobility and radio models, *etc*. [15,40]. Channel measurements of the test bed are incorporated into the simulator. The measurements are analyzed to provide accurate models for both the average path losses around the body and, more importantly, the temporal variation behavior of the channel. Log-normal and custom path loss models based on the measurements are available. The radio module consists of cc1000 and cc2420 transceiver parameters along with the support of several modulation schemes, including frequency shift keying (FSK), phase shift keying (PSK) , differential binary phase shift keying (DBPSK) and differential quadrature phase shift keying (DQPSK). Baseline MAC (IEEE 802.15.6 draft proposal) is partially available. The advantages of Castalia include specific BAN’s channel models to realistically simulate BAN. Finally, with regards to BAN and BBN, MAC and routing protocols are proposed in [55,56,57], respectively. The baseline IEEE 802.15.6 MAC is enhanced by using models with temporal variations and average path losses based on real-body measurements [56]. Whereas, the other studies do not use BAN-specific channel and propagation models; further, all of these studies lack a realistic radio link and mobility models.

With all the above-mentioned key features, Castalia seems an attractive choice. However, it has certain key limitations with respect to diverse mobility modeling, the IEEE 802.15.6 standard compliance protocol and communication stack, as well as the lack of BBN modules. In mobility modeling, only the line mobility (forward and backward movement) model is available, which is a very basic mobility pattern. Complete IEEE 802.15.6 PHY and MAC are not implemented.

### 2.3. Summary

To summarize, there are a number of general simulators; however, BAN and BBN require specific channels, radio links, mobility models, protocols and algorithms, which are not completely covered by most of these simulators. With regards to BAN-specific simulators, there are a number of specific contributions as highlighted (in Section 2.2.2): the frameworks of OMNET++ and the WSNet extensions through physical simulator PyLayers and co-simulation platform HarvWSNet. All of these contributions are in bits and pieces, and there is no single all-in-one package that can provide a complete and realistic BAN simulator. One of the main limitations with these simulators is that BAN-specific standards’ (*i.e.*, IEEE 802.15.6 or IEEE 802.15.4j) compliance is not available, and consequently, there is no BAN-specific benchmark. Please note, in this study, we have used the WSNet simulator, because it is an open source and modular wireless network simulator. Furthermore, this simulator was shown to provide more realistic and comprehensive radio link and channel modeling in comparison to existing simulators (e.g., NS-2, GloMoSim, JiST/SWANS, *etc*.), especially regarding interference management [58]. This is one of our motivations, to provide a standard compliant benchmark simulator, and therefore, the WSNet simulator is extended to be an IEEE 802.15.6-compliant simulator. In addition to that, as body-to-body networks are a new emerging extension of BAN, the existing simulators do not contain specific features, such as data dissemination strategies [59], appropriate routing protocols [60], multi-standard compliance networking and coexistence strategies [19,61] and standard specific MAC design [16,62]. These are the key additions to existing WSNet simulators to provide (all-in-one) realistic platforms for BAN and BBN.

Finally, please note that, most of the developed modules and corresponding repository will be freely available to the wider research community at [63].

## 3. Proposed Simulator

**Figure 1:**
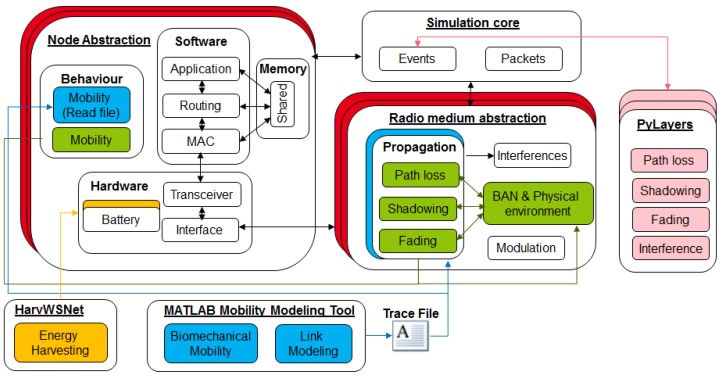
Adaptation of the WSNet simulator to BAN/body-to-body networks (BBN) networks.

The WSNet simulator has been adapted to BAN and BBN contexts in order to fulfill the upper layers’ requirements (*i.e.*, MAC, network and application layers) as shown in Figure 1. Importantly WSNet is adapted with respect to the multi-scale dynamics of inter-BAN multi-link channels.

In this paper, we describe two approaches computing the received signal strength (RSS) in which we focus on joint mobility and propagation models *i.e.*, deterministic bio-mechanical modeling (blue approach) and semi-deterministic stochastic mobility modeling (green approach). Once the RSS is computed according to the considered propagation models and parameters, the propagation modules return the evaluated RSS to the WSNet simulation core, which, according to the current concurrent transmissions, will evaluate the corresponding SINR (signal-to-interference plus noise ratio), BER (bit error rate) and PER (packet error rate). The BER between the transmit node *i* and receive node *j* is computed based on the current SINR level at time *t* (*i.e.*, $SINR_{ij}^{t}$), and the considered physical layer characteristics (e.g., data rates and modulation schema) are as follows:

(1) $$\begin{array}{c} \begin{array}{c} BER_{ij}^{t}=\left\{\begin{array}{cc} 0.5\times e^{-Eb/No} & \text{DBPSK}\\ Q(\sqrt{4^{Eb/No}}\times sin\left(\sqrt{2}\times \pi /4\right)) & \text{DQPSK} \end{array}\right. \end{array} \end{array}$$

where Eb/No is the energy per bit-to-noise power spectral density ratio in dBm which is computed based on the current SINR level as:

(2) $$Eb/No\left[dB\right]=SINR_{ij}^{t}\left[dB\right]+10\times log_{10}\left(BW/R\right)$$

where BW is the bandwidth in Hz and *R* is the data rate in bps.

Finally, the PER between the transmit node *i* and receive node *j* can be evaluated as:

(3) $$PER_{ij}=1-{\left(1-BER_{ij}^{t}\right)}^{n}$$

where *n* is the packet length in bits and $BER_{ij}^{t}$ is the corresponding BER.

### 3.1. Semi-Deterministic (Stochastic) Radio Link and Mobility Modeling

In this subsection, we briefly describe the implementation of the BAN mobility, the BAN environment and the channel models used to realistically reproduce on-body intra-BAN communications and body-to-body channels to model the interference generated by one BAN on another. Channel models are based on an extensive measurement campaign carried out in a classical office environment at a frequency of 2.48 GHz and recorded with a four-port vector network analyzer VNA. The measurements included the presence of up to four people, three antenna on-body positions (head, belt and wrist) and multiple mobility scenarios. It has to be noted that channel realizations for each BAN are generated independently from the others.

**Figure 2:**
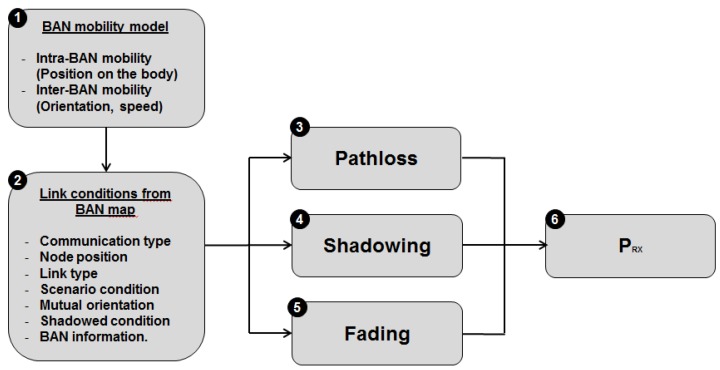
Radio link modeling approach.

As shown in Figure 2, six main steps are considered to model the radio link between a transmitter and a receiver.

The first step consists of extracting some important deterministic information (*i.e.*, positions, direction, speeds) from the mobility model, at every simulation iteration. These information are treated by the BAN environment in the second step to define link conditions between the transmitter and the receiver thanks to the BAN and physical simulation module. The channel is modeled through three modules in the WSNet simulator: path loss (Step 3), shadowing (Step 4) and fading (Step 5). Link conditions are the input parameters of body-to-body and on-body channel models to select the configuration of each model and generate realistic traces. Depending on the considered link condition, the parameters of the propagation model are selected in a dynamic way during the simulation. Once the received signal strength is computed according to the considered propagation models and parameters, the propagation modules return the evaluated RSS to the WSNet simulation core, which, according to the current concurrent transmissions, will evaluate the corresponding SINR, BER and PER.

An essential feature of our simulation is our semi-deterministic approach instead of the statistical approach. The mobility model provides for each realization some important deterministic information (*i.e.*, positions, relative orientations, speeds, obstructions, *etc.*), which are treated as input parameters by body-to-body and on-body channel models to generate realistic traces (*i.e.*, with consistent mobility patterns) as a function of time. For example, on-body channels can adapt their models based on human walking according to the BAN speed. Body-to-body channels can dynamically take into account BAN and physical environment information (e.g., the mutual orientations, the (non-)line of sight conditions and the room to which the other BAN belongs) for interfering links.

#### 3.1.1. Mobility Model

The mobility model is a mixture of group mobility (inter-BAN) and entity mobility (intra-BAN). The inter-BAN mobility model is based on the reference point group mobility model (RPGM) in which the logical center describes the group movement, while the nodes are moving around this logical center. Each logical center or BAN position could follow any kind of mobility model. In our simulations, we have arbitrarily defined a random way point model in which each BAN moves to a newly-chosen destination with a newly-chosen speed and keeps confined in the two rooms. The mobility model provides for each iteration some important deterministic information (*i.e.*, positions, direction, speeds), which is treated by the BAN environment to be the defined link condition, the input parameters of body-to-body and on-body channel models to generate realistic traces.

Concerning the intra-BAN mobility, the human walking movement is directly taken into account in the channel model through the auto-regressive model implementation for time correlation and through the Cholesky model implementation for inter-link correlation. Nevertheless, in order to be in accordance with the inter-BAN mobility model, each sensor on the BAN will use the logical center position and a position-specific drift, and the channel model has been adapted to take the speed into consideration.

#### 3.1.2. BAN Environment

**Figure 3:**
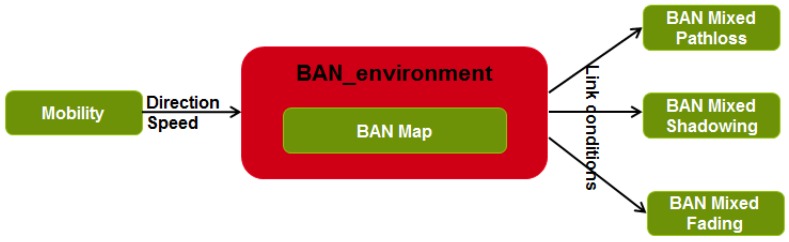
Central position of the BAN environment between the mobility and propagation modules.

The module called “BAN and physical environment” aims at modeling the environment in which wireless nodes are deployed and the attenuation resulting from the propagation of radio signals depending on the radio link conditions and the scenarios. As shown in Figure 2 and Figure 3, this module has a central position.

The BAN and physical environment module exchanges information with the mobility model (e.g., positions, directions, speeds, obstruction) and with BAN propagation models (e.g., link conditions) in order to select the corresponding channel model in our database.

**Figure 4:**
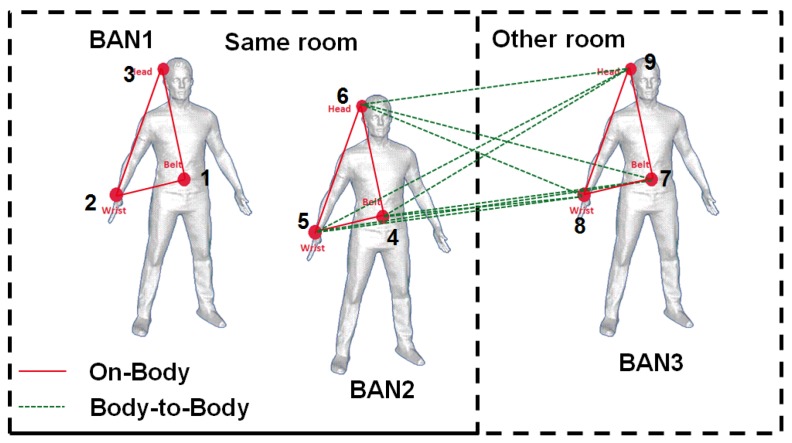
Scenario of several BANs collocated in the same or adjacent rooms.

As shown in Figure 4, we assume that a BAN is formed by three nodes: belt, head and wrist. A multi-BAN network can be seen as multiple star networks. The belt can be considered as the collecting node and/or the coordinator. Some walls and rooms have also been defined in order to evaluate indoor scenarios.

The role of the BAN and physical environment module is to determine the link conditions between the transmitter and the receiver. The link conditions have several characteristics:

- Communication type: off-body, on-body, body-to-body, off-to-off.
- Node position on the body: belt, wrist, head, off.
- Link type: belt-to-head, belt-to-wrist, belt-to-belt, *etc*.
- Scenario condition: same room, other room.
- Mutual orientation and body-shadowed condition for inter-BAN models.
- BANs’ information, such as BAN ID, BAN size, *etc*.

**Figure 5:**
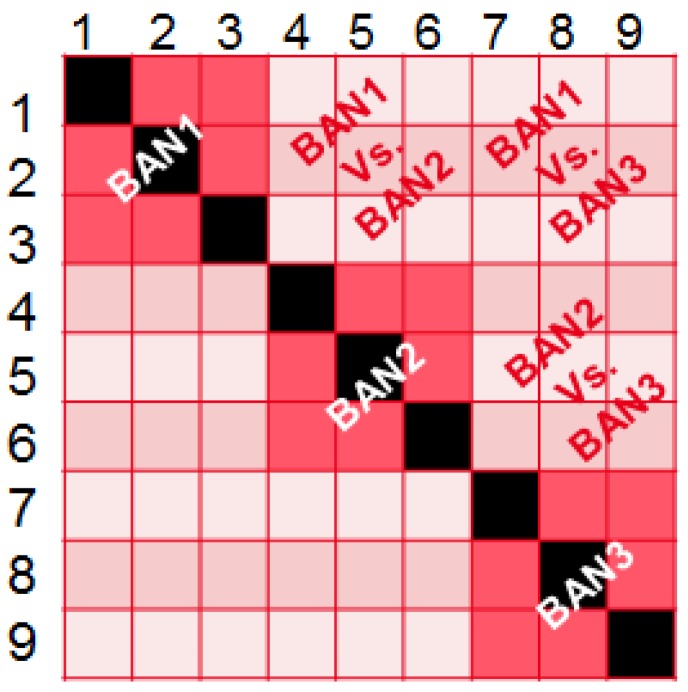
Link condition matrix for each link (Tx-Rx).

In Figure 5, the three dark blocks represent intra-BAN communications respectively for BANs 1–3 and the light blocks’ inter-BAN communications. According to the link conditions, we will select the corresponding channel model parameters in our database.

#### 3.1.3. On-Body Channel Model

On-body channels are classically investigated on a scenario-based approach [38], as body shadowing and human mobility are the factors that have an impact to the greatest extent on their characteristics. In this sense, a scenario is the combination of the specific Tx and Rx antenna locations on the body and the human body mobility condition. The implementation of the on-body channel model is rather simple and mostly relies on the generation of time-correlated long- and short-term fading with given distribution parameters. Moreover, as long-term fading of on-body channels strongly depends on body shadowing, it is relevant to investigate their inter-link correlation, which can significantly affect, for instance, wrist-related links, where the arm movement effects may create correlated links. On the other hand, the average channel gain only depends on the on-body antenna position, as shown in Table 1.

The temporal correlation of both short- and long-term fading is enforced by applying an auto-regressive (AR) method, which linearly combines outputs at previous times with a temporally-uncorrelated input random process. Its formulation to derive an output *y* at a time instant *t* is the following:

(4) $$y\left(t\right)=-\sum_{k=1}^{p}a_{k}y\left(t-k\right)+x\left(t\right)$$

where *p* is the order of the AR model, *x* is the input process and $a_{k}$ are the weights that minimize the variance of prediction error. Table 2 provides the AR coefficients for on-body links. The implementation requires a large number of significant figures to preserve the convergence of the AR method.

Long-term fading inter-link correlation is implemented through a Cholesky factorization of the correlation matrix.

(5) $$\left[\begin{array}{c} x_{c}^{\left(hb\right)}\left(t\right)\\ x_{c}^{\left(hw\right)}\left(t\right)\\ x_{c}^{\left(bw\right)}\left(t\right) \end{array}\right]=\left[\begin{array}{c} x^{\left(hb\right)}\left(t\right)\\ x^{\left(hw\right)}\left(t\right)\\ x^{\left(bw\right)}\left(t\right) \end{array}\right]\cdot C$$

where C is the 3 × 3 Cholesky factorized matrix, $x_{c}$ is the correlated process and the indices of the superscripts identify the different type of channel ( h stands for head, b for belt and w for wrist).

In our implementation, the Cholesky factorized matrix is:

(6) $$C=\left[\begin{array}{ccc} 1 & 0.21 & 0.51\\ 0 & 0.9777 & 0.228\\ 0 & 0 & 0.8294 \end{array}\right]$$

The intra-BAN short-term fading is implemented with a Rice distribution, whose parameters have been derived from measurements, as shown in Table 3. In addition, we assume a coherence time of 20 ms for all links, *i.e.*, short-term fading is considered constant over such a period of time.

**Figure 6:**
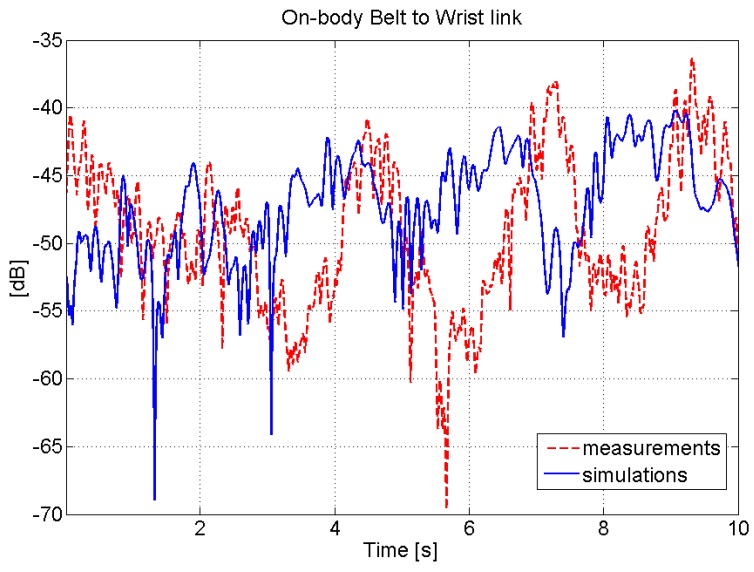
Comparison between measured and implemented belt-to-wrist on-body channel.

Figure 6 shows a comparison between the measured and implemented belt-to-wrist on-body channel.

#### 3.1.4. Body-to-Body Channel Model

**Figure 7:**
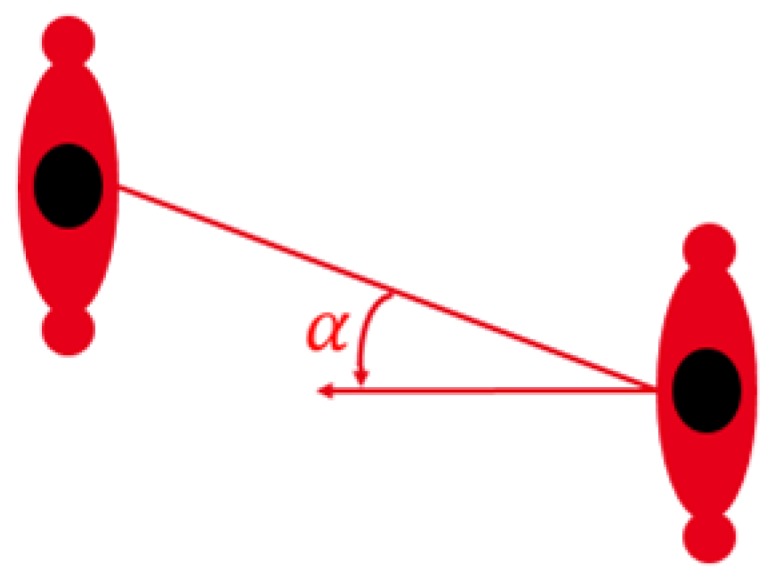
Reciprocal orientation angle.

In B2B communications, the human body itself plays an important role, and when compared to other types of channels, body shadowing has a major influence on the channel characteristics. The result is that, for a given type of channel, the characteristics strongly depend on the reciprocal position of the bodies where the terminals are mounted. In [64], a comprehensive stochastic channel model that accurately predicts narrowband B2B channel behavior by exploiting the awareness of the mutual spatial position of the two terminals has been presented. In this model, the channel depends on the type of link, but it also is a function of the distance (*d*) and the mutual orientation (*α*), defined as in Figure 7, between the two bodies in the following way:

(7) $$G\left(d,\alpha \right)=G_{0}\left(\alpha \right)-10n\left(\alpha \right)\log_{10}\left(\frac{d}{d_{0}}\right)$$

**Figure 8:**
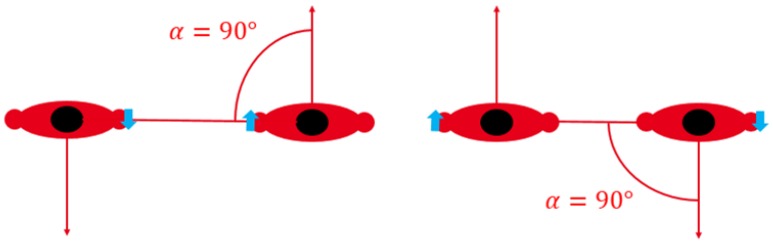
LOS position on the left and body-shadowed position on the right with the same reciprocal orientation angle.

It is also important to note that for wrist-related links, the definition of *α* is not sufficient to describe body shadowing condition. As depicted in Figure 8 for a given angle *α*, the link may be obstructed by the bodies or not. Therefore, an additional parameter has to be included to distinguish between those two possibilities.

In Table 4, some examples of path loss parameterization are provided for head-to-head and belt-to-belt links to show the impact of body shadowing through body orientation. In the first case, there is no significant impact, as propagation was only minorly affected by the on-body antenna position, while in the second one, it is of major importance to include the body shadowing effects produced by the torso. A detailed model will be available in [64].

**Figure 9:**
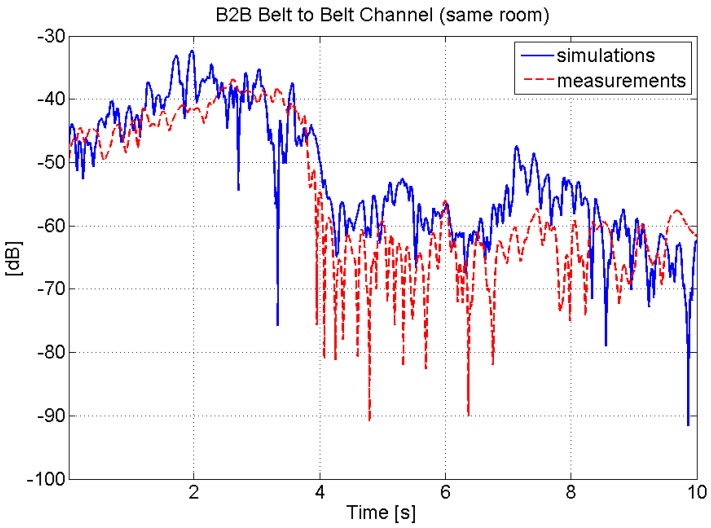
Comparison between measured and implemented belt-to-belt body-to-body (B2B) channel.

Short-term fading has also been shown to have a non-negligible distance and mutual orientation dependence in some of the links, and this issue has to be taken into account to generate an accurate realization of this process [65]. Long-term fading is, instead, implemented with an AR model for temporal correlation, in accordance with what has been done for the on-body links. In this case, no inter-link correlation is implemented. This is because most of the contribution to inter-link correlation is implemented by default by generating consistently the distance and mutual orientation-dependent channel gain. Figure 9 shows an example of the comparison between the measured and implemented belt-to-belt B2B channel, where it is also evident how the model is able to reproduce the short-term fading variations over time induced by the different reciprocal positions of the bodies.

### 3.2. Deterministic (Bio-Mechanical) Radio Link and Mobility Modeling

Wireless body-to-body networks (BBN) are a relatively new dimension of BAN in which multiple bodies interact and share certain information. In this section, we will explain various cross-layer components of the BBN system, which have a direct impact on the performance evaluation of BBN. The accurate mobility, path loss and channel models are essential to gain more insight into the performance of wireless communication stacks under real deployment and operating assumptions [58,66,67]. This is especially true in the context of BANs and BBNs, whose radio channels might undergo harsh multi-path fast fading and time-varying slow fading due to human body shadowing effects [68]. To that end, we consider in this work the intra-BAN biomechanical mobility and radio link models, which were recently introduced in [66], and we extend them to handle the inter-BAN case.

#### 3.2.1. Intra-/Inter-BANs’ Biomechanical Mobility Modeling

Modeling the mobility and posture behaviors of real human bodies is a complex task. One solution consists of exploiting real-time motion capture data and coupling them with geometrical transformation and analysis techniques to properly investigate the performance of BANs and BBNs under different mobility scenarios (e.g., walking, running, exercising, *etc.*).

In this regard, first of all, a bio-vision hierarchical (BVH) file is utilized, which extracts the skeleton to obtain the markers’ locations/positions. The BVH file consists of two parts: the skeleton part and motion data. The skeleton part of BVH describes the hierarchy and initial pose of the skeleton. The motion part contains the rotation and translation of skeleton joints. The root of the BVH skeleton is always zero, whereas all other non-root joints have three Euler angle rotation data of joints. Then, we need to calculate the rotation matrix and translation matrix of every joint relative to its parent joint. More details regarding biomechanical modeling can be found in [69,70].

The above biomechanical modeling and transformation help to introduce space and time variations into the IEEE 802.15.6 proposed channel models to make them more dynamic and realistic.

**Figure 10:**
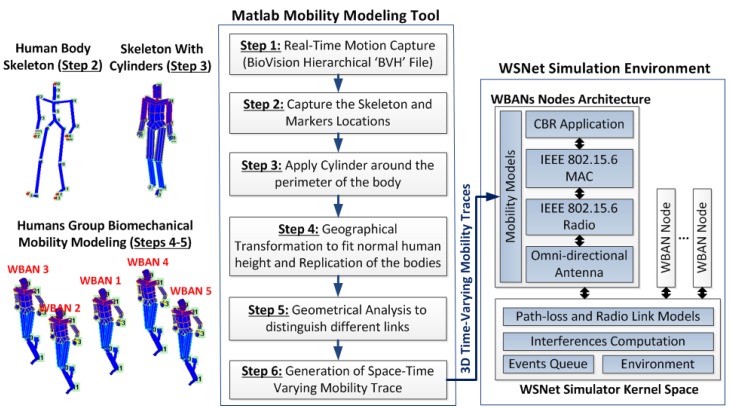
Joint biomechanical, group mobility and radio link modeling for BANs and BBNs.

As shown in Figure 10, our proposed intra- and inter-BANs’ mobility modeling works based on six main steps:

Step 1: real motion capture measurements, which contain the actual human mobility traces according to different mobility scenarios (e.g., walking, running, *etc.*), are extracted into our MATLAB mobility modeling tool [66].

Step 2: The complete human body skeleton is captured from the input motion capture measurements, which consist of a set of markers (*i.e.*, the joints between the different parts of the body) and segments (*i.e.*, the body parts). These markers provide the dynamic distances among all of the locations over time. An example of a human body skeleton is shown in Figure 10.

Step 3: In order to properly model the human body parts (e.g., arms, torso, head, legs, *etc.*), cylinders are applied around the different segments of the human body skeleton. This is an important step to take into account for the body shadowing effects on the performance of radio links; which can either be in the direct LOS or NLOS condition.

Step 4: Geographical transformations are then applied in order to scale the dimensions into normal human height and width. Moreover, the determined human body is replicated in configurable numbers of other human bodies in order to enable the simulation of complex and highly dynamic inter-BAN scenarios.

Step 5: Geometrical analysis is thus applied in order to determine the types of all of the available links (e.g., LOS or NLOS, intra- or inter-BANs) and during the whole trace duration. Exact link types during mobility are evaluated by checking the intersection of the cylinders between all of the links. If a link intersects with a cylinder, then the link is declared as NLOS; otherwise, it is in the LOS state.

Step 6: Finally, space-time-varying links and mobility traces are generated and stored in an external file, which ultimately can be fed into the WSNet packet-oriented simulation environment [58] to enable the realistic performance evaluation of high level communication protocols.

#### 3.2.2. Intra-BAN and Inter-BAN Channel Models

Once the space-time varying links and mobility traces are properly generated for a given mobility scenario, channel models can be applied in order to assess the performance of radio links. The IEEE 802.15.6 standard has proposed various channel models, including the *CM3* (body surface-to-body surface) and CM4 ( body surface-to-external) models.

CM3-Aproposed a log-normal distributed channel model. During the experimentation, the transmitter antenna was placed at the waist, and a receiver antenna was placed on different parts of the body, including the head, ear, shoulder, wrist, waist, leg and ankle. The path loss model was derived using a regression line through least square fitting for both frequency bands at narrow-band (*i.e.*, 900 MHz and 2450 MHz) and is expressed as:

(8) $$PL\left(d\right)\left[dB\right]=a\cdot log_{10}\left(d\right)+b+N$$

where *a* and *b* are the coefficients of the linear fitting, *d* is the distance between (transmitter and receiver in mm), *N* is the normally-distributed random variable with standard deviation *σ*, which has different values based on the frequency bands and the environment, *i.e.*, hospital room or anechoic chamber, and the values are presented in Table 5 [71]. However, it was shown that these models provide only basic distance-based path loss without any time-varying effects and correlation features [66]. The enhanced IEEE 802.15.6 path loss models as proposed in [66] utilize dynamic mobility and obtain space and time-variations.

For the case of CM3-B, the transmitter was worn at approximately shoulder height at two different positions. The receiver was placed directly below the transmitter at seven positions separated by 10 cm covering the range from the shoulder to the knees. Six different locations were considered inside an office room, and at each of the locations, 49 points were arranged at the height of a 7×7 square grid. The separation distance between each array element is 6 cm for 2.45 GHz and 16 cm for 900 MHz. The resulting path loss model is a combination of the exponential function of the distance and average indoor attenuation, which is expressed as:

(9) $$PL\left(d\right)\left[dB\right]=-10\;log_{10}\left(P_{0}\;e^{-md}+P_{1}\right)+N$$

where $P_{0}$ is the average loss close to the antenna and will depend on the type of antenna, *m* represents the average decay rate in dB/cm for the surface wave traveling around the perimeter of the body, *d* is the distance in centimeters, $P_{1}$ is the average attenuation of components in an indoor environment radiated away from the body and reflected back towards the receiving antenna and *N* is the random Gaussian variable with zero mean and 3.6-dB variance ($\sigma_{P}$) [72], measured at different body and room locations. This parameter depends on variations in the body curvature and antenna radiation properties at different body locations, and their typical values are presented in Table 6.

Both above-explained IEEE channel models (*i.e.*, CM3-A and CM3-B) are static models. In order to practically utilize these models, dynamic space and time variations are injected through bio-mechanical model. To highlight the full potential of the deterministic approach, we exploit many mobility scenarios that contain space and time-varying link types, as shown below.

**Figure 11:**
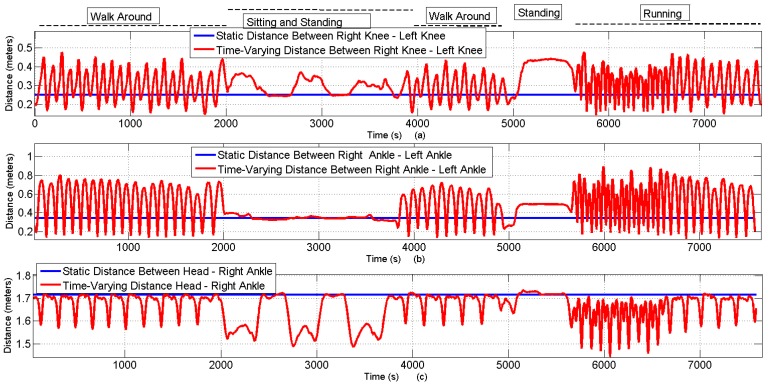
Comparison of static and time-varying distances under different scenarios over time. (**a**) Time varying distances between the right knee and left knee; (**b**) Time varying distances between the right ankle and left ankle; (**c**) Time varying distances between the head and right ankle.

Figure 11 shows distance variations during the sequence of five different scenarios, including *“walk around”*, *“sitting and standing”*, *“standing”* and *“running”* over 60 s with the time resolution of 8.3 ms. The comparison of time-varying *versus* static distances against (38×38) link positions is obtained; however, only three link examples (*i.e.*, *“right knee-left knee”*, *“right ankle-left ankle”* and *“head-right ankle”)* are shown. The important temporal variations are visible during the walking and running scenarios.

**Figure 12:**
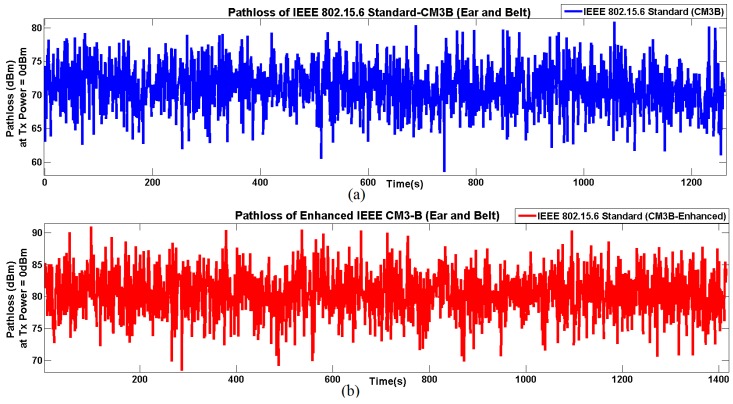
Path loss comparison of CM3-B *versus* enhanced CM3-B at 2450 MHz. (**a**) Pathloss of IEEE 802.15.6 standard CM3-B between (Ear and belt); (**b**) Pathloss of Enhanced IEEE 802.15.6 standard E-CM3-B between (Ear and belt).

After obtaining the time-varying distances, these are used to accurately model the channel behavior. For example, static *versus* time-varying path loss comparisons of CM3-B and Enhanced Channel Model (E -CM3-B) are shown in Figure 12. As an example, the results are shown for a 2450-MHz frequency under walking scenarios. Further, a selected link (*i.e.*, *“ear-belt”*) is shown to be coherent with the semi-deterministic approach presented in Section 3.1. However, Table 7 shows the most interesting link types, where the deterministic approach is far more realistic than the IEEE 802.15.6 proposed channels [73]. It is important to note that the E-CM3-B model provides higher and more accurate time variations, which are due to two main reasons. First, time-varying mobility traces with accurate distances have enabled more accurate path loss results in comparison with static CM3-B, as can be seen in Figure 12. Second, “LOS/NLOS” link types are identified, and specific path loss modifications in the enhanced models are as follows:

- If the link type is LOS, then we use Equations (8) and (9) only with the dynamic distances to compute the path loss.
- Else, if the link type is NLOS, then we use Equations (8) and (9) with the dynamic distances to compute the path loss. Further, an additional NLOS factor is used as an add-on to the model. As an example, on average, 13% extra of the factor (which was determined experimentally in [74]) should be added in the case of NLOS links. This factor has been added in every NLOS link in the enhanced models.

Table 7 shows three different links (*i.e.*, “right knee-left knee”, “right ankle-left ankle” and “right toe-left toe”), which contains both space (*i.e.*, distance) and time variations (*i.e.*, mobility). Statistical results, including mean, standard variation, as well as correlation coefficients, are presented for four channel models under two frequencies. It is quite obvious that the mean variations among the enhanced and standard channel models are very close, and in terms of standard deviations, there are slight differences. However, the major difference is in the time correlation. It can be observed that the enhanced models with dynamic distances keep track of certain patterns, which can be periodic, such as shown in Table 7 and in Figure 12. It is to be noted that the results are presented for the “*jogging/running*” scenarios shown in the latter part of Figure 11, as it provided higher mobility. Though for the *“walking”* scenario, the differences between the comparisons of the correlation coefficient are relatively less.

Regarding the correlation coefficient, the numbers of links were under observations during the simulations, in particular some other mobile links, such as *“hands”*, or *“wrists”* *versus* *“chest”*, *“lowerback”* and *“hips”*. Most of these links provide less space variations as the movements of the hands or wrists are much less in comparison to the movements of one foot from another, and therefore, it is observed that these links provide (on average) a 5% to 15% difference in the correlation coefficients. Another important observation is that CM3-B/E-CM3-B (*i.e.*, the exponential models) shows much higher correlation than the CM3-A/E-CM3-A (lognormal distributed models). One of the reasons is that the CM3-B model remains almost flat at distances higher than 0.35 m because of the dominating impact of multipath fading, whereas E-CM3-B keeps track of high mobility and temporal variation and, hence, provides much higher correlation coefficients.

However, in the case of a radio link of type inter-BAN, *i.e.*, the two nodes *i* and *j* are located on different BANs, the corresponding path loss is computed as [38]: $PL\left(d_{ij}\right)=G\left(d_{0}\right)+10\times n\times log_{10}\left(d_{ij}/d_{0}\right)+F$; where $G(d_{0})$ is the channel gain at the reference distance, $d_{0}$ is the reference distance, which is equal to 1 m, *n* is the path loss exponent factor and *F* is the fading. Typical values (validated experimentally) for these components are provided in [38].

## 4. Performance Evaluation

To compare the two approaches presented above fairly, there are a number of constraints and limitations. For example, with the semi-deterministic approach, there are limitations to the number of nodes per BAN and mobility patterns. Whereas, the deterministic mobility approach is not limited by the number of nodes, but mainly by the mobility patterns and duration, which are imported from motion capture systems. With regards to channel models, in particular to incorporate the body shadowing and fading, the semi-deterministic approach characterizes these factors and includes their impacts separately, whereas the deterministic approach is inclusive, but does not characterize these factors separately. It should be noted that a common application scenario and the setup for the evaluation of the two approaches are therefore limited to the above-mentioned constraints.

### 4.1. Simulation Setup and Scenario

A packet-oriented network simulator, WSNet [58], is used (as shown in Figure 1) to evaluate the BAN and BBN application scenarios. WSNet contains various models for wireless sensor networks, wireless local area networks and *ad hoc* networks. However, previously, it did not contain BAN-specific modules. Therefore, we have enhanced the simulator (with the focus on IEEE 802.15.6 standard compliance) to accurately model body area networks using enhanced channel models, an accurate radio link and mobility models (as explained earlier in Section 3.2 and Section 3.1).

In order to replicate the following simulation results, Table 8 presents the details of each model being used along with the configuration parameters and their values. It is important to mention that, for the deterministic approach, any specific scenario depends on the mobility patterns and the corresponding trace files. These trace files are imported in the simulator [18]. We can use diverse external mobility traces (including, swimming, running, walking, sitting, standing, *etc.*). However, to compare the results with the semi-deterministic approach, we limit ourselves to only walking.

However, we have three nodes, located at the belt (the coordinator Node 0), ear (Node 1) and right wrist (Node 2), and mainly, we compare the results for these three locations. It is important to mention that for the deterministic approach, we improve the accuracy of the IEEE 802.15.6 standard channel model by introducing dynamic distances, space and time variations and link types (line of sight, non-line of sight and time-varying periodic links); for example, for NLOS, we add 13% additional path loss according to [75]. To highlight this accuracy, we have presented a few time-varying links (such as ankle, knee and toe), and their correlation behavior is illustrated in Table 7. Finally, if the links are stable (*i.e.*, LOS with fixed distances), then the deterministic approach is the same as the IEEE 802.15.6 standard.

The simulation settings are as follows: at the application layer, two sensor nodes located at the ear and right wrist and one coordinating node (located at the belt) are considered. Every node generates a packet at 100-ms intervals of a size of 256 bytes of payload. From the application layer, every packet is parsed into the MAC layer where the carrier sense multiple access (CSMA/CA) protocol with priorities using a state machine is implemented. The priority level of the data is set to two. The backoff mechanism is followed exactly as proposed in the IEEE 802.15.6 standard (*i.e.*, for every odd backoff, the contention window size is doubled), where maximum backoff and re-transmissions are set as five and four, respectively. Further, the immediate acknowledgment policy is adopted. The overhead of the MAC and physical layers has also been considered according to the IEEE 802.15.6 and the chosen physical layer. In this paper, we will focus on differential quadrature phase shift keying (DQPSK) modulation with the highest data rate (*i.e.*, 971.4 kbps). However, more detailed results on varying parameters can be found in [62].

The simulation setup is based on Version 3.0, which is an up-to-date version of the WSNet simulator. By using all of the above-explained models and parameters in Table 8, WSNet’s XML configuration files are generated for the simulations. The simulations are run for 100,000 packets transmission, and the 95% confidence interval is considered.

### 4.2. Results

The simulation results are presented for three performance metrics, this includes the packet delivery ratio (PDR), packet delay and energy consumption. Along with the two presented approaches, we also consider IEEE 802.15.6 channel models.

**Figure 13:**
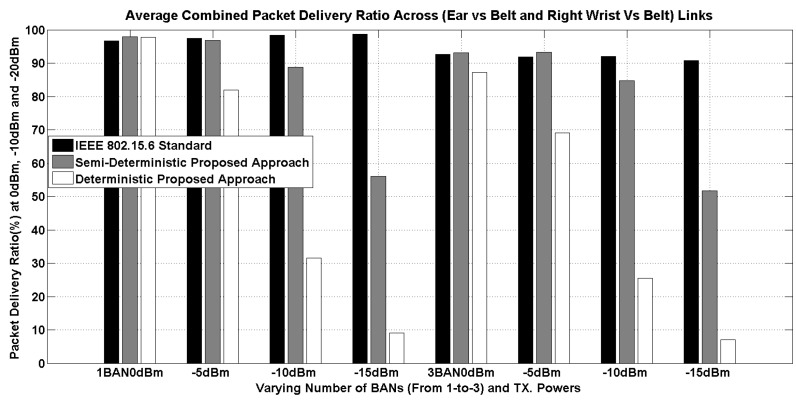
Average packet delivery ratio at varying transmission power for one and three BANs (having three nodes per BAN).

First of all, the results of PDR are presented as shown in Figure 13. It can be noticed that the IEEE 802.15.6 standard has almost no impact on the performance, even by reducing the transmission power to −15 dBm. To understand this behavior, Table 9 shows the average received power for ear-belt and right wrist-belt links. We can clearly see that CM3-B (IEEE channel model) has a significant difference in the actual received power at varying transmission power levels, and it is not always constant. However, since the radio sensitivity (*i.e.*, −85 dBm being used from the IEEE 802.15.6 standard) is much higher than the received power, therefore CM3-B has a higher packet delivery ratio, even at −15 dBm. Now, the question of the slight increase in PDR, while reducing the transmit power, is mainly due to the distributed MAC layer. In the CSMA/CA MAC, there is always randomness, and this is visible in the IEEE CM3-B results. Whereas, the enhanced CM3-B has a significant difference, and it is much closer to the radio sensitivity, even starting from −5 dBm. As a result, the PDR is much lower, as can be seen in Figure 13. Consequently, E-CM3-B is more realistic.

While comparing the results between the IEEE 802.15.6 channel model and the enhanced IEEE 802.15.6 channel model, Our deterministic approach is more pessimistic; the best performance is achieved at 0 dBm, whereas the PDR constantly decreases with reduced transmission power. A similar trend can be seen for the case of multiple BANs. It is to be noted that additional path loss factors (as mentioned in Section 4.1) make the enhanced version more realistic; especially for time varying links, since it provides the dynamic distance with reference to the coordinator which, is not the case in the IEEE 802.15.6 proposed channel models. Further, this has been previously validated specifically against time varying links in [66]. The variations of the PDR results in the deterministic approach while moving from one BAN to three BANs is 6% to 7%. However, the impact of the transmission power variation from 0 dBm to −15 dBm is significant in enhanced CM3-B (*i.e.*, proposed deterministic approach).

With regards to the PDR results of the semi-deterministic approach, it is more optimistic than the deterministic approach. It can be observed that, with reduced transmission power (*i.e.*, −10 dBm), the PDR is nearly 8% to 10% lower than at 0 dBm both for one BAN, as well as three BANs. In general, while moving from one BAN to three, there is a slight decrease in PDR (almost 4%), as can be observed in Table 10. However, at −15 dBm, the PDR is reduced to about 50%. To conclude, the performance results of PDR are not realistic in the IEEE 802.15.6 proposed channel models, and the selection of transmission power needs to be done wisely.

**Figure 14:**
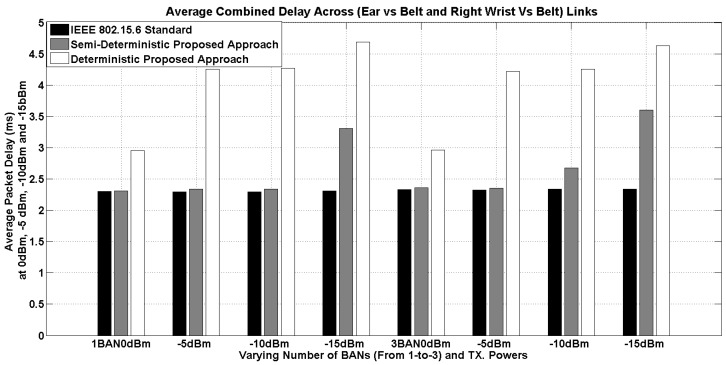
Average end-to-end packet delay at varying transmission power for one and three BANs (having three nodes per BAN).

The results of the average end-to-end packet delay are presented in Figure 14. The delay is computed based on the actual packet delay and the propagation delay. For example, a data packet considering the maximum payload in the IEEE 802.15.6 standard (*i.e.*, 256 bytes) with DQPSK modulation at 971.4 kbps and MAC and PHY headers takes 2.16 ms.

It can be observed that the delay between the IEEE 802.15.6 proposed CM3-B and semi-deterministic approach is closely matched until −10 dBm. However, at −15 dBm, the semi-deterministic approach has almost a 1-ms higher delay. Please note that, at 0-dBm transmission power, for either one or three BANs, the results are almost comparable at millisecond granularity, and mainly, the difference is in the order of microseconds (μs). Whereas, the deterministic approach has the highest delay among the three approaches. On average, it has a 1-ms higher delay, which continues to increase at lower transmission power levels. Finally, the detailed results are available in Table 10.

**Figure 15:**
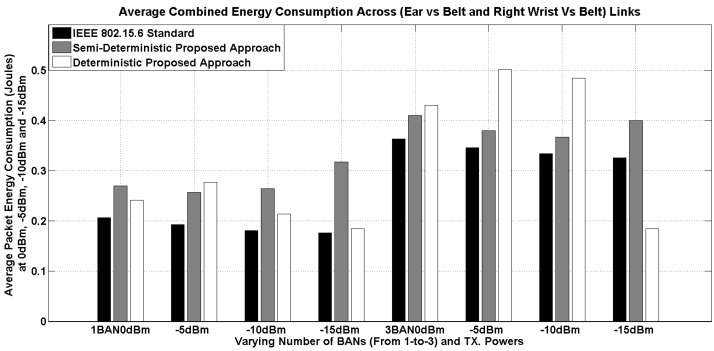
Average energy consumption at varying transmission power for one and three BANs (having three nodes per BAN).

The results of the average energy consumption are presented in Figure 15. The energy consumption for each transmitted packet is calculated as follows,

(10) $$E_{packet}=T_{packet}\times 3_{Volts}\times I_{mA}$$

where $T_{packet}$ is the packet duration in ms, which is based on the effective packet length and is obtained from real propagation time in the simulator, as explained above. The different current consumption values are considered from TI’s cc2420 radio transceiver. For example, for transmission, (17.4, 14, 11, 9) mA is used against the power levels (*i.e.*, (0, −5, −10, −15) dBm). For the reception and idle modes, 19.7 mA and 0.426 mA are used, respectively. Whereas, for the sleep mode, 0.02 mA is used. The energy consumption is estimated by considering a battery of three volts.

For varying transmission power, we have, 17.4 mA (at 0 dBm) and 11 mA (at −10 dBm) current consumption from the cc2420 transceiver. For the receive, idle and sleep states, the current levels are constant, irrespective of transmission power. Therefore, overall, the impact from the power variation is only from the transmission power and the time being spent in each state. Please note that at lower transmission power levels, the main impact is from the idle duration, which is five- to 10-times more compared to the combined transmission and reception duration of the CSMA/CA MAC protocol.

While comparing the energy consumption results, generally, our proposed semi-deterministic approach has higher energy consumption. Under one BAN, the results are comparable between the two proposed approaches until −10 dBm of transmission power; however, at −15 dBm, the semi-deterministic approach has one to two Joules more energy compared to the deterministic approach.

To summarize, it is important to carefully select the transmission power level; apparently, most of the radio transceivers have nearly flat power profiles after −10 dBm, and it does not help to improve the performance. On the contrary, it enhances the delay and reduces the energy efficiency.

## 5. Discussion and Conclusions

Human assistance and wearable technologies, such as BANs, are emerging as an important part of daily life. These information and communication technologies (ICT) have not only helped to provide innovative healthcare solutions, but also are able to significantly reduce healthcare spending around the world. In this regard, body-to-body networks are a relatively new dimension and have been emerging rapidly in recent years.

To model the emerging wearable applications, realistic performance evaluation is critical. This can be achieved and validated through extensive cross-layer simulation. Currently, the existing simulations platforms are limited in the realistic BAN and BBN features and models. In this paper, we present two approaches, semi-deterministic and fully deterministic, to accurately model the BAN and BBN channel models, radio link and mobility models. The semi-deterministic approach is able to generate link-correlated and time-varying realistic traces (*i.e.*, with consistent mobility patterns) for on-body and body-to-body shadowing and fading, including body orientations and rotations, by means of stochastic channel models. The full deterministic approach is particularly targeted to enhance the IEEE 802.15.6 proposed channel models by introducing space and time variations (*i.e.*, dynamic distances) through biomechanical modeling. In addition, it helps to accurately model the radio link by identifying the link types and corresponding path loss factors for LOS and NLOS conditions. This approach is particularly important for links that vary over time due to mobility.

To conclude, there is a clear trend between the two proposed approaches. In the semi-deterministic approach, we propose stochastic models of body fading and shadowing based on measurement campaigns. This approach is more realistic than the deterministic approach and generates link-correlated and time-varying realistic traces mapped on the mobility patterns. Nevertheless, this approach is limited in terms of node positions and the application scenario. On the other hand, the deterministic approach is more suitable to daily life applications with a larger panel of mobility scenarios and a higher number of node positions on the body. It directly enhances the IEEE 802.15.6 standard (*i.e.*, static model) through dynamic mobility patterns, especially for periodic time-varying links.

In the future extension, we will extend the simulator to support networking protocols and more BAN- and BBN-specific mobility models. In addition, some specific application scenarios along with mobility patterns will be integrated.

## Figures and Tables

**Table 1:** Average gain for each on-body link.

On-Body Link	Gain (dB)
**Head-to-Belt**	−48.2
**Head-to-Wrist**	−53.9
**Belt-to-Wrist**	−50.0

**Table 2:** Table of auto-regressive model weights for on-body channels.

AR Coefficient	Belt-to-Wrist	Belt-to-Head	Head-to-Wrist
$a_{0}$	−1.9734512929	−2.0672207748	−2.0268577284
$a_{1}$	0.8052924686	0.9814623443	0.9057458068
$a_{2}$	0.1558380462	0.1007066836	0.1273802520
$a_{3}$	0.0297438008	0.0105872912	0.0178147987
$a_{4}$	0.0047875128	0.0004463117	0.0012784956
$a_{5}$	−0.0004256897	−0.0013910969	−0.0018884276
$a_{6}$	−0.0010198892	−0.0021916391	−0.0026983122
$a_{7}$	0.0029269547	−0.0022352955	−0.0015016341
$a_{8}$	0.0258211963	0.0030632551	0.0118456028
$a_{9}$	−0.0494859747	−0.0231942330	−0.0310823183
$\sigma_{E}$	7.651762983 $\times 10^{-7}$	9.260825677 $\times 10^{-7}$	9.281826210 $\times 10^{-7}$

**Table 3:** Rice parameters of short-term fading for each on-body link.

Short-Term Fading	*μ*	*σ*
**Head-to-Belt**	0.7661	0.4286
**Head-to-Wrist**	0.8407	0.3365
**Belt-to-Wrist**	0.8688	0.2677

**Table 4:** Examples of parametrization of the path loss in a single-room (SR) or two-room (2R) scenario for head-to-head and belt-to-belt links.

	Path Loss Exponent (n)	$G_{0}$ (dB)
**Head-to-Head (SR)**	1.28	−41.5
**Head-to-Head (2R)**	0.94	−46.9
**Belt-to-Belt (SR)**	−0.007α+1.84,0≤α≤90	−0.19α−29.5,0≤α≤100
	−0.027α+3.64,90≤α≤130	−0.55α+6.5,100≤α≤120
	0.011α−1.3,130≤α≤180	0.06α−66.7,120≤α≤180
**Belt-to-Belt (2R)**	1.7	−0.068α−41

**Table 5:** Channel model for the narrow band (*i.e.*, CM3-A).

Path Loss Model	Hospital Room	Anechoic Chamber
(Parameters)		
a	6.6	29.3
b	36.1	16.8
$\sigma_{N}$, dB	3.8	6.89

**Table 6:** Channel model for the narrow band (*i.e.*, CM3-B).

Path Loss Model (Parameters)	Values at 900 MHz	Values at 2.45 GHz
$P_{0}$, dB	0.6512	0.0026
m, dB/cm	48.7212	46.0931
$P_{1}$, dB	$1.1519^{-6}$	$7.4683^{-8}$
$\sigma_{P}$, dB	3.2	3.6

**Table 7:** Statistical analysis of the IEEE 802.15.6 channel models and enhanced proposed channel models using the deterministic approach.

Link Type	Statistics	CM3-A		E-CM3-A		CM3-B		E-CM3-B	
		900 MHz	2450 MHz	900 MHz	2450 MHz	900 MHz	2450 MHz	900 MHz	2450 MHz
Right	Mean	53.48	61.74	55.85	62.75	68.36	80.08	66.17	79.36
Ankle-Left	Standard Deviation	5.50	3.89	6.12	4.01	2.75	3.61	6.32	4.77
Ankle	**Correlation Coefficient**	**0.06**	**0.06**	**0.25**	**0.13**	**0.05**	**0.06**	**0.78**	**0.39**
Right	Mean	51.67	60.80	52.48	61.30	62.62	79.01	63.50	78.32
Knee-Left	Standard Deviation	5.40	3.72	5.76	3.86	2.80	3.64	7.54	5.01
Knee	**Correlation Coefficient**	**0.07**	**0.07**	**0.10**	**0.06**	**0.05**	**0.06**	**0.85**	**0.48**
Left	Mean	53.30	61.69	55.99	62.69	67.95	80.04	65.64	78.80
Toe-Right	Standard Deviation	5.23	3.66	6.16	4.00	2.75	3.59	7.69	5.72
Toe	**Correlation Coefficient**	**0.06**	**0.06**	**0.28**	**0.12**	**0.06**	**0.07**	**0.83**	**0.56**

**Table 8:** Models and their corresponding configurations. DQPSK, differential quadrature phase shift keying.

**Common/Shared**	**Configuration**	**Values**
**(Models)**	**Parameters**	
Application Layer	destination ID, data transmission interval,	Coordinator, 100 ms,
(Constant bit rate (CBR)	size, start time	310 bytes, 0 s
MAC (IEEE 802.15.6	User priority, BAN size,	2, 3
CSMA/CA with ACK)	sleep management, ACK type	1, immediate
Modulation (DQPSK)	Highest data rate	971.4 Kbps
Radio (IEEE 802.15.6)	Tx power, channel, sensitivity	0 to 15 dBm, 0, −85 dBm
Noise	AWGN	−95 dBm
Antenna		Monopole
Interference		Orthogonal
**Different**	**Configuration**	**Values**
**(Models)**	**Parameters**		
Path loss	IEEE 802.15.6: CM3-B	2450 MHz, Table 6
	enhanced IEEE 802.15.6 (E-CM3-B)	2450 MHz,
	path loss-BAN-mixed	2450 MHz,
Mobility	IEEE 802.15.6: Static	Fix distances (*i.e.*, 0.26 m and 0.34 m)
	Enhanced IEEE 802.15.6 Dynamic:	file-time-varying: space and time
	walking, running, standing, *etc.*	varying distances, LOS/NLOS factors
	Semi-deterministic:	mobility-BAN group
	Dynamic (walking)	
**Additional**	**Configuration**	**Values**
**(Models)**	**Parameters**		
Shadowing	Semi-deterministic	Shadowing-BAN mixed
Fading	Semi-deterministic	Fading-BAN mixed

**Table 9:** Average received power for ear and belt (with distance equal to 0.26 m) and right wrist and belt (0.334 m).

Link	Ear-Belt		Right Wrist-Belt	
Tx Power	CM3-B	E-CM3-B	CM3-B	E-CM3-B
0	−52.65	−80.0843	−52.02	−79.9807
−5	−57.69	−85.7168	−56.83	−85.6790
−10	−62.66	−90.4281	−62.03	−89.3149
−15	−67.65	−94.8671	−67.03	−94.2420

**Table 10:** PRR, energy consumption and latency under varying Tx power and BANs for different channel and mobility models. It can be noticed that the granularity of the variations in delay and energy consumption is $10^{-6}$ (*i.e.*, μs) and $10^{-3}$ (*i.e.*, mJ) respectively.

Performance	Tx Power	BAN	IEEE 802.15.6	Proposed Deter-	Proposed Semi-
Metrics	(dBm)	(nbr)	Channel CM3-B	ministicApproach	Deterministic Approach
PRR (%)	0	1	96.65	97.64	97.74
		3	92.66	87.25	93.12
	−5	1	97.48	81.89	96.87
		3	91.88	69.04	93.32
	−10	1	98.43	31.56	88.76
		3	91.95	25.56	84.80
	−15	1	98.62	09.11	56.04
		3	90.82	6.98	51.72
Latency (ms)	0	1	2.3040	2.3080	2.9530
		3	2.3320	2.3620	2.9600
	−5	1	2.9660	2.3370	4.2550
		3	2.3270	2.3535	4.2160
	−10	1	2.9880	4.2660	2.3370
		3	2.3380	4.2550	2.6773
	−15	1	2.3060	4.6890	2.3087
		3	2.3370	4.6310	3.5988
Energy (J)	0	1	0.2070	0.2415	0.2700
		3	0.3630	0.4303	0.4101
	−5	1	0.1932	0.2769	0.2574
		3	0.3461	0.5014	0.3800
	−10	1	0.1813	0.2138	0.2647
		3	0.3341	0.3669	0.4841
	−15	1	0.1758	0.1843	0.3175
		3	0.3258	0.1846	0.4000
